# Will I get dementia? – Symptoms and worries of individuals affected by subjective cognitive decline

**DOI:** 10.1002/alz.086835

**Published:** 2025-01-03

**Authors:** Franziska Kiene, Helmut Hildebrandt, Mandy Roheger

**Affiliations:** ^1^ Carl von Ossietzky Universität Oldenburg, Oldenburg Germany; ^2^ Hospital Bremen‐Ost, Bremen Germany

## Abstract

**Background:**

Subjective cognitive decline (SCD) is a condition, where individuals report persistent decline of cognitive abilities, even though this decline is not detectable by neuropsychological screenings. Individuals with SCD are at a higher risk of suffering from mild cognitive impairment (MCI) and Alzheimer’s disease (AD) in the future. It is important to better understand SCD to develop prevention measures, before a transition from a possible preclinical stage to MCI and AD.

**Method:**

To get an insight into SCD symptomatology, focus group discussions with individuals affected by SCD were conducted. A comprehensive neuropsychological assessment was done beforehand. Focus groups were audio‐recorded, transcribed and analyzed with MAXQDA. Content analysis was performed using inductive and deductive approaches.

**Result:**

4 group discussions with 16 participants in total (11 female, 5 male) were conducted. Although, a decline was reported for each cognitive domain (attention, memory, orientation, visuocognition, speech, executive functioning, learning), participants especially reported a decline of abilities typically impaired in AD: word‐finding, episodic memory and orientation, with declining word‐finding being amongst the first noticed symptoms, most frequently expressed and heavily affecting, as it leads to social withdrawal due to difficulties in conversations. Participants also frequently mentioned a declining prospective memory, short term memory and working memory. Individuals with SCD are especially concerned about a progression of decline and suffering from AD in the future. 9 participants already sought medical help due to SCD.

**Conclusion:**

SCD as a possible preclinical stage of AD needs to be further researched. Although, individuals with SCD are experiencing impairments in their everyday life, they are not receiving support within the German health care system, as often no objectifiable cognitive impairments are detected by simple screening tools. Therefore, results of this study will be used to develop a questionnaire for measuring SCD. The aim is to develop an ambulatory SCD questionnaire to categorize the subjective feeling of cognitive decline and to identify subjects which should be assessed thoroughly.

